# Ge on Si waveguide mid-infrared absorption spectroscopy of proteins and their aggregates

**DOI:** 10.1364/BOE.398013

**Published:** 2020-07-28

**Authors:** Vinita Mittal, George Devitt, Milos Nedeljkovic, Lewis G. Carpenter, Harold M. H. Chong, James S. Wilkinson, Sumeet Mahajan, Goran Z. Mashanovich

**Affiliations:** 1 Optoelectronics Research Centre, University of Southampton, Southampton, SO17 1BJ, United Kingdom; 2 Institute for Life Sciences, University of Southampton, Southampton, SO17 1BJ, United Kingdom; 3 School of Electronics and Computer Science, University of Southampton, Southampton, SO17 1BJ, United Kingdom; 4 School of Chemistry, University of Southampton, Southampton, SO17 1BJ, United Kingdom

## Abstract

Specific proteins and their aggregates form toxic amyloid plaques and neurofibrillary tangles in the brains of people suffering from neurodegenerative diseases such as Alzheimer’s and Parkinson’s. It is important to study these conformational changes to identify and differentiate these diseases at an early stage so that timely medication is provided to patients. Mid-infrared spectroscopy can be used to monitor these changes by studying the line-shapes and the relative absorbances of amide bands present in proteins. This work focusses on the spectroscopy of the protein, Bovine Serum Albumin as an exemplar, and its aggregates using germanium on silicon waveguides in the 1900–1000 cm^−1^ (5.3–10.0 µm) spectral region.

## 1. Introduction

The mid-infrared (MIR) part of the electromagnetic spectrum is known as the molecular fingerprint region because most molecules have fundamental vibrations in this region, making it useful to specifically identify molecules and sensitively quantify concentrations through absorption spectroscopy. MIR spectroscopy is a well-established non-destructive analytical technique for the qualitative and quantitative analysis of molecules present in any phase of matter, making it attractive for applications including analytical chemistry, astronomy, forensics, food, pharma, biomedical imaging and sensing. These applications are not only technologically significant but also contribute to a safe, secure and healthy society [1,2]. MIR spectroscopy is most commonly implemented using Fourier transform infrared spectroscopy (FTIR) in transmission, but conventional instruments are often bulky and the thin cuvettes appropriate for aqueous samples can become easily blocked when applied to real samples. To overcome the liquid (especially aqueous) sample handling problem, FTIR is often combined with attenuated total reflection (ATR) spectroscopy, where light is confined within an ATR element made of an infrared transparent bulk material such as germanium, zinc selenide or diamond. Absorption takes place in the evanescent field present on top of the ATR element due to (frustrated) total internal reflection, where the liquid sample is in contact with the ATR element surface. Since the thickness of an ATR element is typically large (mm scale), there are usually only a few discrete reflections along the (few cm) ATR element length, so that a large sample volume is required to cover the entire ATR element and even then the sensitivity to absorption for a given element length, is low. Thin film optical waveguides supported on a solid substrate can be used as a form of ATR element, but in that case their thickness and width usually have micron-scale dimensions, leading to a very strong and, in the case of monomode waveguides, continuous evanescent field at their surface [3,4]. This leads to similar sensitivity as in the case of ATR but for much shorter lengths. Given the narrow lateral confinement in waveguides small sample volumes can be interrogated and highly compact chip-based devices can be fabricated. While waveguide thicknesses and widths are typically micron-sized, their lengths can range from microns to tens of cm and, for example, spiral waveguides can offer long lengths on small chip areas [4,5]. Waveguides can thus be tailored to the analyte and achieve strong sensitivity to absorption with small sample volumes. Optical waveguides can be mass-manufactured by standard fabrication processes compatible with CMOS technology, which reduces the cost of production. Their small size and the possibility of on-chip integration of multiple devices offers the potential for low-cost, compact and portable multi-analyte detection instruments. Diamond and zinc selenide (ZnSe) ATR crystals are routinely used in FTIR measurements along with the Ge crystal and have a wider transparency window in the MIR. Both diamond and ZnSe have refractive index (n) close to 2.4 whereas Ge has n∼4. A thin film waveguide requires a suitable substrate that is transparent in the region of interest as well as have a lower refractive index than waveguide core material. Silicon dioxide substrate is the most appropriate choice in terms of its mature technology and low refractive index (n∼ 1.4), however, it absorbs strongly beyond λ = 3.7 um, hence cannot be used in spectroscopy of proteins to detect amide bands. There are few alternative substrates available such as fluorides and sulfides, but more research is needed to establish low-loss waveguide platform using these material systems. We have chosen Ge on Si (n∼3.5) waveguide system for several reasons. First, Ge can be epitaxially grown on Si and is transparent in the protein sensing region of interest and has low optical losses [6,7]. Si technology is mature and compatible with CMOS, therefore, mass-manufacturing of thousands of devices on a single wafer is easy and cost-effective. Microfluidics and electronic read out circuitry can be easily integrated with silicon chips that could lead to potential for developing affordable and multiplexed integrated silicon photonics sensors for a number of clinical applications. It was also found that the shape or structure of BSA protein is not altered on binding to the Ge surface [8].

Proteins are crucial for understanding the physiological and psychological state of living beings and may be found in human blood, urine, sweat etc. In many neurodegenerative disorders such as Alzheimer’s disease, the pathological hallmark is the aggregation of specific proteins. Aggregation leads to changes in the secondary structure or conformation of the proteins as they evolve from monomers to oligomers and ultimately fibrils (polymers) [9]. The oligomers are considered to be the toxic species [10]. Therefore, the onset and progression of such diseases could be revealed by detecting the conformational changes of these specific proteins and their oligomers (which are generated in the human brain) in the cerebrospinal fluid. The MIR absorption in proteins occurs predominantly due to vibrations of the polypeptide backbones referred to as amide bands. All three of these amide bands lie in the spectral region between 1900 and 1000 cm^−1^, and can be observed on a Ge on Si waveguide as shown by us recently [8]. This article focuses on detailed study of the conformation changes in aqueous BSA protein solution, as an exemplar, as it aggregates from its monomeric to oligomeric and fibrillar state by monitoring the line shape and position of the individual components of amide I band such as α-helices, β-sheets and random turns. The novelty of this work lies in the detection of conformation changes in aqueous protein solution as opposed to the dried sample despite the strong water absorption in the MIR. The common additional step of drying of samples takes time and the drying procedure affects the workflow and the results [11]. A very significant disadvantage of measuring dried rather than aqueous samples is that proteins change their conformation on drying [12,13], creating inaccuracies in their interpretation, so it is crucial to measure proteins in biofluids in their aqueous state. Waveguide measurement of aqueous protein samples in the MIR combined with high power light sources such as QCLs has the potential to detect low concentration of protein in aqueous medium with less than 1µL of sample volume used for the measurements.

Raman spectroscopy is a complementary technique to IR spectroscopy. Whilst also measuring vibrational modes of molecules, the selection rules for Raman and IR differ, resulting in distinct spectral fingerprints for a given molecule. Whilst Raman spectroscopy relies on inelastic scattering, IR spectroscopy relies on absorption, and is therefore an order of magnitude more sensitive. On the other hand, water is a weak scatterer, but strongly absorbs in the IR region, making Raman desirable for biological samples. However, spontaneous Raman spectroscopy demonstrates weak signals due to low quantum efficiency. Typically this means pre-concentration or specialized techniques need to be used for obtaining spectra. Hence, given that MIR absorption has high cross-sections and thus gives higher signals, and provided the device can be engineered to mitigate water absorption, biofluid analysis in the MIR becomes an attractive application for especially with on-chip detection.

## 2. Materials and methods

### 2.1 Waveguide fabrication and characterization

**Fig. 1.**
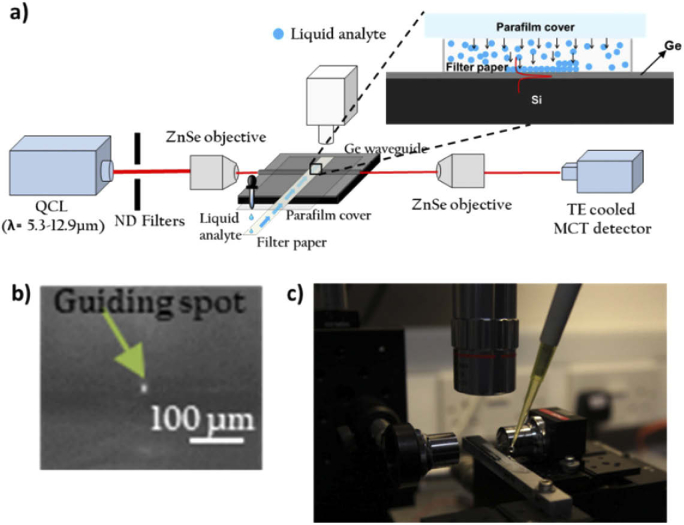
a) Schematic of the experimental apparatus (The cross-section of the waveguide is magnified), b) Photograph of the apparatus showing liquid from the pipette is introduced on the tail of the filter paper placed on GOS waveguide and c) IR image of cross-section of the GOS waveguide channel showing confinement of light.

A 6 inch wafer of 3 µm thick germanium on silicon (GOS) from IQE Silicon Compounds Ltd was used to fabricate the waveguides. The Si substrate is n-type in <100> orientation with the resistivity between 1 - 10 ohm.cm. Strips 20 µm wide were patterned in the germanium film using lithography and the Ge was fully etched to the silicon substrate using fluorine chemistry in an inductively coupled plasma (ICP) tool to form the waveguides. The end facets were prepared using ductile dicing [14]. Waveguide spectroscopy was carried out using the apparatus shown in Fig. 1(a). A quantum cascade laser (QCL) (Block Engineering Inc.) tunable from 1900-800 cm^−1^ (wavelength of 5.3–12.9 µm) was used as the light source. The pulsed laser is Transverse Magnetic (TM) polarised resulting in the electric field polarized perpendicular to the waveguide substrate surface, with line width 1 cm^−1^and pulse width between 50-500nsec, pulse repetition rate of upto 1MHz and duty cycle of upto 5%. The average power of the laser is between 0.5-10mW. Two zinc selenide (ZnSe) microscope objective lenses were used for input and output coupling. A thermoelectrically-cooled mercury cadmium telluride (MCT) detector (VIGO System) was used to measure the signal from the collection objective lens. The signal from the MCT detector was recorded on a computer which also controlled the tuning of the QCL, and a software package (LaserTune), was used to process the spectra.

The waveguides are multimode and were found to be guiding light over the full range from 5.3–12.9 µm [8]. A mode image of the 20 µm wide waveguide captured on an IR camera is shown in Fig. 1(b) guiding at λ = 12.9 µm. As a simple microfluidic structure, a strip of filter paper was used to introduce aqueous analytes onto the surface of the waveguide and to define the evanescent absorption pathlength. A parafilm cover was used on top of the filter paper to avoid sample evaporation. A portion of waveguide is magnified in Fig. 1(a) to show the cross-section of the waveguide where liquid analyte travels to the waveguide surface through the pores of the filter paper. It has been established that the presence of the filter paper on the surface of waveguide does not itself significantly alter the optical transmission [2]. Figure 1(c) shows a camera image of the apparatus while taking the measurements. It shows the waveguide sample kept on a stage between two lenses and a pipette carrying the aqueous solution of proteins is used to drop the liquid on the tail of the waveguide from where it travels through capillary action to the waveguide that is aligned with the lenses and the detector. The lens seen on the top of the waveguide sample is the microscope objective. It was also confirmed that the waveguide surface of Ge does not denature or significantly alter the secondary structure of BSA proteins when measured at different concentrations [8].

### 2.2 Protein sample preparation for AFM and MIR measurements

A 900 µM Bovine Serum Albumin (BSA) (Sigma Aldrich) solution of the monomeric protein was prepared in 10 mM HCl (pH = 3, prepared in double-distilled water). The monomer solution was stored in an Eppendorf tube in a water bath maintained at 65°C for 10 min to form agglomerates known as oligomers. For AFM measurements, the fresh protein samples were diluted 1/1000 and then allowed to bind to a mica substrate for two minutes, which was then washed 4 times in double distilled water and allowed to dry. They were imaged using a Digital Instruments Multimode IV AFM system operated in tapping mode. Aluminum coated probes with a resonance frequency of 320 kHz and force constant of 42 N/m were used for all images (Nanoworld, POINTPROBE NHCR). Probes were autotuned using Nanoscope III 5.12r3 software before use. Images shown are representative of the sample and were taken at random points on the sample with a scan rate of 1Hz-2Hz and 512 samples per line/512 lines per image. For details on AFM sample preparation, please refer Ref. [9]. For MIR absorption spectroscopy, the proteins samples stored in Eppendorf tube were introduced onto the waveguide surface via the filter paper fluidics by using a pipette

## 3. Results

**Fig. 2.**
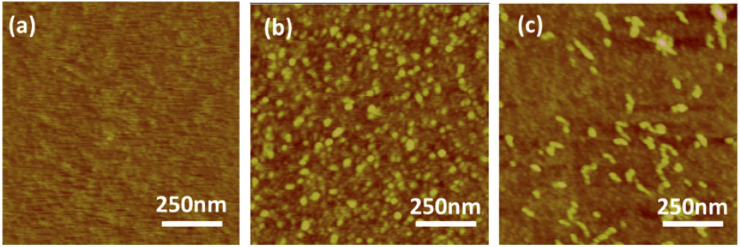
AFM images of (a) monomers and (b) oligomers and c) fibrils of BSA protein of area 1 µm x 1 µm. and height 15 nm.

Atomic force microscopy (AFM) is a topographical imaging technique that enables structures to be resolved at the nanoscale based on weak interactions between the sample and a probe. Therefore, AFM was used to track the morphological evolution of BSA during aggregation to confirm the formation of higher order aggregation species, specifically oligomers and fibrils. Figure 2(a) and (b) shows 1 µm x 1 µm AFM images of monomers and oligomers of BSA protein, respectively. The images clearly show that monomers aggregate to form bigger assemblies known as oligomers as seen in Fig. 2(b), which subsequently elongated into fibrils as seen in Fig. 2(c). The vibrational modes of each species were subsequently probed using MIR spectroscopy to allow any changes in internal structural composition to be determined.

**Fig. 3.**
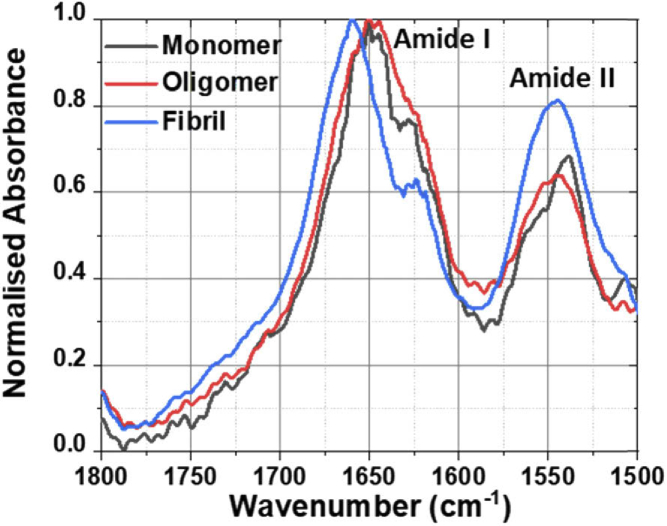
Waveguide absorption spectra of aqueous BSA solution measured on Ge on Si waveguide showing the amide I and II bands for monomers, oligomers and fibrils.

To establish a reference measurement for the protein spectroscopy, first, pure water was pipetted onto the tail of a 2 mm wide and 4 cm long strip of filter paper and allowed travel across the chip to the waveguide and the transmitted power spectrum was recorded. After cleaning the waveguide, BSA monomer solution was pipetted onto a fresh strip of filter paper of a similar width, and the transmitted power spectrum was again recorded. 10 sample scans were recorded and averaged separately for both reference and protein spectra. The scan resolution was 1 cm^−1^ To calculate the normalised absorption spectrum, the contribution of the solvent (buffer) was taken out by dividing the sample spectrum by that of the buffer. The transmitted power spectrum from the monomer sample, T_m_, was divided by the reference transmitted power spectrum from the buffer solution, T_b_, to determine the change in transmission due to the BSA,, and the resultant monomer absorbance spectrum was obtained by taking the log of T_b_/T_m_, according to the Beer-Lambert law. The resultant data was normalized to unity and then smoothed using 10-point adjacent averaging. The oligomer sample absorption spectrum was measured and processed in the same way. The normalized smoothed absorption spectra for the monomer and oligomer solutions are shown in Fig. 3 and both the amide I band at 1650 cm^−1^ and amide II band at 1540 cm^−1^ are clearly observed. The amide I band results largely from amide carbonyl stretching (C = O), whereas the amide II band results from a combination of N-H bending and C-N bending and is weak (and often not present) in Raman spectra without enhancement. The amide II band is somewhat sensitive to secondary structure, although interpretation is more complex than for the amide I band [15].

**Fig. 4.**
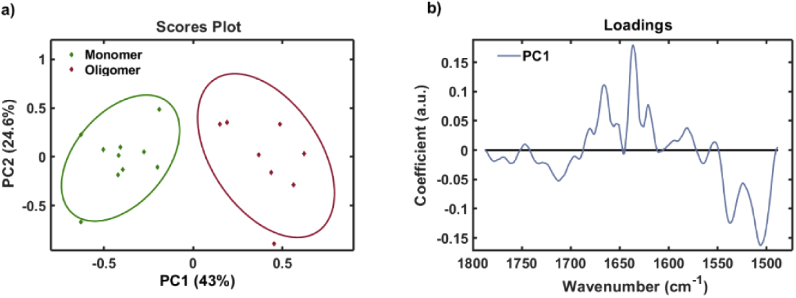
Principal component analysis of BSA monomer and oligomer amide I spectra. a) 2-dimensional PCA scores plot showing distribution of monomer spectra (red diamonds) and oligomer spectra (blue diamonds) across PC1 and PC2 axes. b) PC1 loadings spectrum highlights the variation responsible for PCA scores.

The detailed shape of the amide I band of proteins is widely used to study their secondary structure or conformation [16–19]. Amide I frequencies can be assigned to α-helix, β-sheets, random coil, β-turns, intermolecular and anti-parallel β-sheets that forms the secondary structure of a protein and have specific absorption frequency. These sub-bands and their absorption frequencies are tabulated in Table 1 according to the literature [16–20]. Both the BSA monomer and oligomer spectrum show an amide I maxima ∼1650 cm^−1^, suggesting a predominantly α-helical conformation, as expected from the crystal structure [21]. While both the monomer and oligomer absorption spectra in Fig. 3 overlap to a great extent, there are some subtle but clear differences. For detection purposes the most critical requirement is the ability to be able to identify oligomers and differentiate them from monomers. Hence, we used Principal Component analysis (PCA) in order to identify variation between the IR spectra for monomers and oligomers (Fig. 4). PCA is an unsupervised method of statistical analysis that reduces data dimensionality by the generation of an orthogonal set of principal components that best describe the variance within the data. PCA has been used to identify spectral changes that represent alterations in protein conformation during aggregation [9]. PCA was performed using the IRootLab [22] plugin (0.15.07.09-v) for MATLAB R2015a. 10 spectra from each class were included in the PCA. All spectra were background subtracted using blank buffer spectra and smoothed using the wavelet denoising function with six decomposition levels (0, 0, 100, 1000, 1000). Spectra were then trimmed between 1788 cm^−1^ and 1488 cm^−1^ and intensities were normalized using the amide I band. Trained-mean centering was applied to the spectra before PCA with a maximum of ten principal components. Cross-validation was performed using the leave-one-out method.

**Table 1.** Assignment of deconvolved components of the amide I band ^16-20^

Wavenumbers (cm^−1^)	Band assignment	Peaks associated with oligomerisation from PCA
1601-1617	Intermolecular β-sheets	1621
1626-1643	β-sheets	1637
1644-1649	Random Coil	
1650-1658	α-helix	
1662-1680	β-turns	1666
1690	Anti-parallel β-sheets	

Soluble oligomeric species that have undergone a structural conversion have been shown to be the toxic intermediates in the aggregation [23]. Figure 3. shows that conformational differences are more subtle between BSA monomers and oligomers than between BSA monomers and fibrils. It may be important to detect such subtle changes in conformation upon the formation of oligomeric proteins to allow the early detection of neurodegenerative disease. In order to better classify monomer and oligomer spectra, we performed PCA on the IR amide I spectra (Fig. 4). The scores plot shows that one principal component (PC1) can be used to distinguish the IR spectra of BSA monomers and oligomers (Fig. 4(a)). The loadings spectrum represents the weights of each variable used for calculation of that principal component, meaning that the loadings contain the source of variation for a given PC. The spectral variation across this PC1 axis is highlighted by the PC1 loadings in Fig. 4(b), with positive peaks corresponding to the positive score associated with oligomer spectra, including β-sheet (1621 and 1637 cm^−1^) and β-turns (1666 cm^−1^). The spectral frequency of individual secondary structure components are assigned according to Table 1. It is well established that the formation of toxic protein aggregates is due in an increase in intermolecular β-sheet structure [17–18,21,23–24], which agrees well with our findings. These observations also agree with Raman shifts observed during BSA oligomerisation that correspond to an increase in β-sheet structure [9,25].

Monomer and oligomer spectra were used for PCA as there were only subtle differences between these spectra, whereas the fibril spectra were clearly distinct. PCA was also able to distinguish monomer and oligomer spectra from fibril spectra, as expected (Refer Supplementary figure S1 in the Supplemental Document).

## 4. Conclusion

In this work, MIR spectroscopy of Bovine Serum Albumin and its aggregates is demonstrated on a GOS waveguide and analyzed in detail. The detection of structural changes in proteins and their toxic aggregates is crucial for early diagnosis of neurodegenerative diseases such as Alzheimer’s disease. In the present work, we have demonstrated, as an exemplar, that changes in the structure of BSA protein and its aggregates can be evaluated by deconvolving the amide I band into its individual components which establishes that waveguide spectroscopy can be used to distinguish monomers from the toxic oligomers by measuring and comparing the conformational content in terms of α-helix and β-sheets. Since monomers and aggregates yields absorption at specific frequencies, aggregates were found to be rich in β-sheets content, which is consistent with the literature. This optical sensing technique enables low-cost, highly sensitive MIR silicon chip-based waveguide sensor that combines the advantages of optics and microfluidics. Such optical biosensors based on silicon photonics offer compact, portable and scalable multi analyte detection sensing where thousands of sensors can be mass-produced on an 8” silicon substrate using microfabrication mass manufacturing, allowing affordable and multiplexed sensors for broader clinical access and efficient population screening. In future work, proof of concept detection of pathological species in artificial cerebrospinal fluid or blood plasma will be studied on this waveguide sensor platform.

## Notes

See Supplement 1 for supporting content.

## References

[1] Seiger M. Mizaikoff B., “Toward on-chip mid-infrared sensor,” Anal. Chem. 88(11), 5562–5573 (2016). 10.1021/acs.analchem.5b04143 27081763

[2] Mittal V. Nedeljkovic M. Rowe D. Murugan G. S. Wilkinson J. S., “Chalcogenide glass waveguides with paper-based fluidics for mid-infrared absorption spectroscopy,” Opt. Lett. 43(12), 2913–2916 (2018). 10.1364/OL.43.002913 29905722

[3] Tran M. Huang D. Komljenovic T. Peters J. Malik A. Bowers J., “Ultra-low-loss silicon waveguides for heterogeneously integrated silicon/III-V photonics,” Appl. Sci. 8(7), 1139 (2018). 10.3390/app8071139

[4] Nedeljkovic M. Penades J. S. Mittal V. Murugan G. S. Khokhar A. Z. Littlejohns C. Carpenter L. G. Gawith C. B. Wilkinson J. S. Mashanovich G. Z., “Germanium-on-silicon waveguides operating at mid-infrared wavelengths up to 8.5 µm,” Opt. Express 25(22), 27431–27441 (2017). 10.1364/OE.25.027431 29092216

[5] Li W. Anantha P. Lee K. H. Qiu H. D. Guo X. Goh S. C. K. Zhang L. Wang H. Soref R. A. Tan C. S., “Spiral waveguides on germanium-on-silicon nitride platform for mid-IR sensing applications,” IEEE Photonics J. 10(3), 1–7 (2018). 10.1109/JPHOT.2018.2829988

[6] Gallacher K. Millar R. W. Griškevičiūte U. Baldassarre L. Sorel M. Ortolani M. Paul D. J., “Low loss Ge-on-Si waveguides operating in the 8–14 µm atmospheric transmission window,” Opt. Express 26(20), 25667–25675 (2018). 10.1364/OE.26.025667 30469665

[7] Chang Y. C. Paeder V. Hvozdara L. Hartmann J. M. Herzig H. P., “Low-loss germanium strip waveguides on silicon for the mid-infrared,” Opt. Lett. 37(14), 2883–2885 (2012). 10.1364/OL.37.002883 22825166

[8] Mittal V. Nedeljkovic M. Carpenter L. G. Khokhar A. Z. Chong H. M. H. Mashanovich G. Z. Bartlett P. N. Wilkinson J. S., “Waveguide absorption spectroscopy of bovine serum albumin in the mid-infrared fingerprint region,” ACS Sens. 4(7), 1749–1753 (2019). 10.1021/acssensors.9b00215 31264410

[9] Devitt G. Rice W. Crisford A. Nandhakumar I. Mudher A. Mahajan S., “Conformational Evolution of Molecular Signatures during Amyloidogenic Protein Aggregation,” ACS Chem. Neurosci. 10(11), 4593–4611 (2019). 10.1021/acschemneuro.9b00451 31661242

[10] Verma M. Vats A. Taneja V., “Toxic species in amyloid disorders: Oligomers or mature fibrils,” Ann. Indian Acad. Neurol. 18(2), 138 (2015). 10.4103/0972-2327.144284 26019408 PMC4445186

[11] Finlayson D. Rinaldi C. Baker M. J., “Is infrared spectroscopy ready for the clinic?” Anal. Chem. 91(19), 12117–12128 (2019). 10.1021/acs.analchem.9b02280 31503460

[12] Moorthy B. S. Iyer L. K. Topp E. M., “Characterizing protein structure, dynamics and conformation in lyophilized solids,” Curr. Pharm. Des. 21(40), 5845–5853 (2015). 10.2174/1381612821666151008150735 26446463 PMC4671836

[13] Odoli C. O. Oduor-Odote P. Arason S., “The influence of lipid content and pretreatment methods on protein conformation in fish (capelin, Mallotus villosus) during smoking and drying,” Food Sci. Nutr. 7(4), 1446–1454 (2019). 10.1002/fsn3.980 31024718 PMC6475805

[14] Mittal V. Aghajani A. Carpenter L. G. Gates J. C. Butement J. Smith P. G. Wilkinson J. S. Murugan G. S., “Fabrication and characterization of high-contrast mid-infrared GeTe_4_ channel waveguides,” Opt. Lett. 40(9), 2016–2019 (2015). 10.1364/OL.40.002016 25927772

[15] Krimm S. Bandekar J., “Vibrational spectroscopy and conformation of peptides, polypeptides and proteins,” Adv. Protein Chem. 38, 181–364, Academic Press (1986). 10.1016/S0065-3233(08)60528-8 3541539

[16] Jung C., “Insight into protein structure and protein–ligand recognition by Fourier transform infrared spectroscopy,” J. Mol. Recognit. 13(6), 325–351 (2000). 10.1002/1099-1352(200011/12)13:6<325::AID-JMR507>3.0.CO;2-C 11114067

[17] Lu R. Li W. W. Katzir A. Raichlin Y. Yu H. Q. Mizaikoff B., “Probing the secondary structure of bovine serum albumin during heat-induced denaturation using mid-infrared fiberoptic sensors,” Analyst 140(3), 765–770 (2015). 10.1039/C4AN01495B 25525641

[18] Nakamura A. Kaneko N. Villemagne V. L. Kato T. Doecke J. Doré V. Fowler C. Li Q. X. Martins R. Rowe C. Tomita T., “High performance plasma amyloid-β biomarkers for Alzheimer’s disease,” Nature 554(7691), 249–254 (2018). 10.1038/nature25456 29420472

[19] Nabers A. Perna L. Lange J. Mons U. Schartner J. Güldenhaupt J. Saum K. U. Janelidze S. Holleczek B. Rujescu D. Hansson O., “Amyloid blood biomarker detects Alzheimer's disease,” EMBO Mol. Med. 10(5), 8763 (2018). 10.15252/emmm.201708763 PMC5938617 29626112

[20] López-Lorente Á. I. Wang P. Sieger M. Vargas Catalan E. Karlsson M. Nikolajeff F. Österlund L. Mizaikoff B., “Mid-infrared thin-film diamond waveguides combined with tunable quantum cascade lasers for analyzing the secondary structure of proteins,” Phys. Status Solidi A 213(8), 2117–2123 (2016). 10.1002/pssa.201600134

[21] Majorek K. A. Porebski P. J. Dayal A. Zimmerman M. D. Jablonska K. Stewart A. J. Chruszcz M. Minor W., “Structural and immunologic characterization of bovine, horse, and rabbit serum albumins,” Mol. Immunol. 52(3-4), 174–182 (2012). 10.1016/j.molimm.2012.05.011 22677715 PMC3401331

[22] Trevisan J. Angelov P. P. Scott A. D. Carmichael P. L. Martin F. L., “IRootLab: a free and open-source MATLAB toolbox for vibrational biospectroscopy data analysis,” Bioinformatics 29(8), 1095–1097 (2013). 10.1093/bioinformatics/btt084 23422340

[23] Cremades N. Cohen S. I. Deas E. Abramov A. Y. Chen A. Y. Orte A. Sandal M. Clarke R. W. Dunne P. Aprile F. A. Bertoncini C. W., “Direct observation of the interconversion of normal and toxic forms of α-synuclein,” Cell 149(5), 1048–1059 (2012). 10.1016/j.cell.2012.03.037 22632969 PMC3383996

[24] Della Porta V. Bramanti E. Campanella B. Tiné M. R. Duce C., “Conformational analysis of bovine serum albumin adsorbed on halloysite nanotubes and kaolinite: A Fourier transform infrared spectroscopy study,” RSC Adv. 6(76), 72386–72398 (2016). 10.1039/C6RA12525E

[25] Devitt G. Howard K. Mudher A. Mahajan S., “Raman spectroscopy: an emerging tool in neurodegenerative disease research and diagnosis,” ACS Chem. Neurosci. 9(3), 404–420 (2018). 10.1021/acschemneuro.7b00413 29308873

