# Supplementary material for: Ge on Si waveguide mid-infrared absorption spectroscopy of proteins and their aggregates
Source: Biomed Opt Express. 2020 Jul 28;11(8):4714–22. doi: 10.1364/BOE.398013 (PMC7449756; doi:10.1364/BOE.398013)
Supplement: Supplementary file 1 [file boe-11-8-4714-s001.pdf]

Supplemental Document

Biomedical Optics EXPRESS

Ge on Si waveguide mid-infrared absorption spectroscopy of proteins and their aggregates: supplement

VINITA MITTAL,[1,*] GEORGE DEVITT,[2] MILOS NEDELJKOVIC,[1] LEWIS G. CARPENTER,[1] HAROLD M. H. CHONG,[3] JAMES S. WILKINSON,[1,2] SUMEET MAHAJAN,[2,4] AND GORAN Z. MASHANOVICH[1]

[1] *Optoelectronics Research Centre, University of Southampton, Southampton, SO17 1BJ, United Kingdom*
[2] *Institute for Life Sciences, University of Southampton, Southampton, SO17 1BJ, United Kingdom*
[3] *School of Electronics and Computer Science, University of Southampton, Southampton, SO17 1BJ, United Kingdom*
[4] *School of Chemistry, University of Southampton, Southampton, SO17 1BJ, United Kingdom*
[*] *V.Mittal@soton.ac.uk*

This supplement published with The Optical Society on 28 July 2020 by The Authors under the terms of the Creative Commons Attribution 4.0 License in the format provided by the authors and unedited. Further distribution of this work must maintain attribution to the author(s) and the published article's title, journal citation, and DOI.

Supplement DOI: https://doi.org/10.6084/m9.figshare.12681797

Parent Article DOI: https://doi.org/10.1364/BOE.398013

Ge on Si waveguide mid-infrared absorption spectroscopy of proteins and their aggregates : supplementary material

VINITA MITTAL[1]*, GEORGE DEVITT[2], MILOS NEDELJKOVIC[1], LEWIS G. CARPENTER[1], HAROLD M. H. CHONG[3], JAMES S. WILKINSON[1,2], SUMEET MAHAJAN[2,4] AND GORAN Z. MASHANOVICH[1]

[1]*Optoelectronics Research Centre, University of Southampton, Southampton, SO17 1BJ United Kingdom*
[2]*Institute for Life Sciences, University of Southampton, Southampton, SO17 1BJ United Kingdom*
[3]*School of Electronics and Computer Science, University of Southampton, Southampton, SO17 1BJ United Kingdom*
[4]*School of Chemistry, University of Southampton, Southampton, SO17 1BJ United Kingdom*
**V.Mittal@soton.ac.uk*

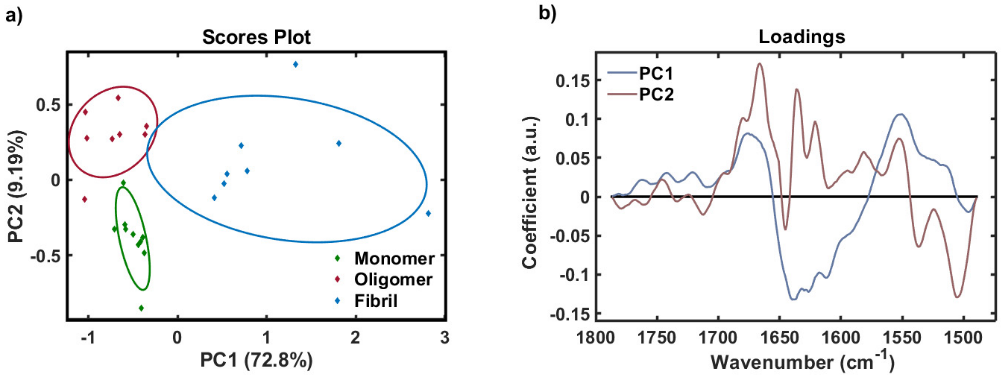

Fig. S1. Principal component analysis of BSA monomer, oligomer and fibril amide I spectra.
a) 2-dimensional PCA scores plot showing distribution of monomer spectra (red diamonds), oligomer spectra (blue diamonds) and fibrils (green diamonds) across PC1 and PC2 axes. b) PC1 and PC2 loadings spectrum highlights the variation responsible for PCA scores.
